# Evaluating Core Psychiatry Training Provisions in Cwm Taf Morgannwg University Health Board Using a Mock GMC Survey

**DOI:** 10.1192/bjo.2025.10314

**Published:** 2025-06-20

**Authors:** Tyler Thomas, Timothy Chan, Kok Keong Leong, Daniel De Silva, Siddhartha Baiju

**Affiliations:** 1 CTMUHB, Cardiff, United Kingdom; 2 CTMUHB, Bridgend, United Kingdom.; 3 CTMUHB, Llantrisant, United Kingdom

## Abstract

**Aims:** This survey aimed to assess the training conditions of psychiatry resident doctors, within Cwm Taf Morgannwg University Health Board (CTMUHB). The survey provided an opportunity for trainees to share their experiences, highlight challenges, and contribute to improving training. Participation was anonymous to ensure confidentiality for sensitive topics such as discrimination.

**Methods:** The survey was developed in collaboration with resident doctors and the College Tutor Committee (CTC). Initial planning took place in May 2024, with survey design and distribution occurring in July 2024. Data collection focused on themes such as induction, rota design, and supervision. Data were cross-referenced with the GMC National Training Survey and HEIW Core Psychiatry Training data for validation. Although our respondent numbers vary vastly from that of the GMC National Training survey, significant proportionate interpretation of concerns raised in CTMUHB were made. Results are currently guiding discussion with the CTC and Health Board executives to implement strategic interventions.

**Results:** Respondents highlighted several concerns, particularly in induction processes, rota design, facilities, and consultant availability. Governance-related issues, such as inadequate escalation pathways, were also evident. 22% of respondents strongly disagreed that they had received all necessary information during induction, compared with just 2% in the national GMC survey. 11% rated their induction as “very poor”. Rota management was another major concern, with 33% of residents expressing dissatisfaction over unfilled rota gaps, which they felt resulted in missed learning opportunities. Additionally, 44% of CTMUHB residents reported working beyond their rostered hours, a figure substantially higher than the national rate of under 12%. Alarmingly, 22% of respondents were unaware of how to raise concerns about their training, indicating a critical gap in reporting mechanisms. Reports of discrimination, burnout, and negative workplace experiences further underscored the need for urgent intervention.

Despite these challenges, positive aspects were noted. All respondents agreed that their educational supervisor was easily accessible, and every trainee received formal feedback. However, 22.2% found this feedback unhelpful. Many residents highlighted teamwork and a supportive work environment as key factors contributing to overall job satisfaction.

**Conclusion:** The Health Board specific findings highlight the need for targeted interventions to improve training conditions. Recommendations include enhancing induction processes, redesigning rota management, increasing consultant availability and improving reporting systems. Addressing workplace discrimination and fostering a supportive environment remain critical priorities. Continued collaboration between resident doctors and the CTC is essential to drive meaningful improvements and ensure a better training experience for future trainees.

